# Stakeholder's Views on the Development of Mobile Application (TechCare) for Patients With First Episode With Psychosis: Qualitative Study

**DOI:** 10.1192/bjo.2024.151

**Published:** 2024-08-01

**Authors:** Zaib un Nisa, Nadeem Gire, Ameer Bukhsh Khoso, Imran B. Cahudhry, Nusrat Husain

**Affiliations:** 1 Pakistan Institute of Living and Learning, Karachi, Pakistan; 2 University of Bolton, Manchester, United Kingdom; 3 Zia ud Din University Hospital, Karachi, Pakistan; 4 University of Manchester, Manchester, United Kingdom

## Abstract

**Aims:**

Psychosis is one of the leading causes of disability. First Episode Psychosis (FEP) significantly impacts the long-term course of the disorder. While a majority of FEP service users show signs of ‘recovery' within 12 months of treatment, the early course involves frequent relapses, with up to 80% relapsing within five years. This elevates the risk of persistent psychotic symptoms, affecting cognitive, social, and occupational functioning. Medication, the core treatment, reduces relapse by 75%, necessitating additional psychosocial treatments. Mobile-based interventions are recognized for meeting families' needs in terms of information, guidance, and support. This paper explores stakeholder views on developing mobile interventions for those experiencing their first psychosis episode.

**Methods:**

This qualitative paper was part of the TechCare app development process in which face-to-face interviews with patients (17), and 4 focus groups with health professionals were carried out. The qualitative interviews and focus groups explored the views of stakeholders on the need for mobile-based treatment, the structure of the application, the content of the application and barriers and challenges were also explored in detail. All the audio-recorded interviews were transcribed and analyzed through a framework approach.

**Results:**

Qualitative analysis revealed three themes. The first theme centers on *stakeholders' views about mobile-based treatment*. Health professionals reported that app-based treatment enhances help-seeking behavior, reduces societal stigma, and aids in managing treatment and activities. The second theme focuses on *suggestions for the Techcare application,* emphasizing logical and easy-to-understand content, with a major focus on crisis management, hallucinations, and psycho-education about symptoms. Participants also highlighted the need for a section providing psycho-education for families. Carers emphasized the necessity of an activity plan in the app, including an activity log for medication management and activities. The third theme delves into *barriers and challenges in app-based treatment*, including difficulty levels and privacy concerns. Stakeholders stressed the importance of content in simple Urdu language for broader understanding.

**Conclusion:**

In conclusion, mobile-based treatment contributes to reducing stigma, increasing awareness about the illness in its early stages, and facilitating the management of functional activities for patients. The insights gathered from stakeholders provide valuable guidance for the development of an effective and culturally sensitive mobile-based intervention for individuals experiencing FEP.

